# A simulation-based evaluation of machine learning models for clinical decision support: application and analysis using hospital readmission

**DOI:** 10.1038/s41746-021-00468-7

**Published:** 2021-06-14

**Authors:** Velibor V. Mišić, Kumar Rajaram, Eilon Gabel

**Affiliations:** 1 Decisions, Operations and Technology Management, Anderson School of Management, University of California Los Angeles, Los Angeles, CA USA; 2 Department of Anesthesiology and Perioperative Medicine, University of California Los Angeles, Los Angeles, CA USA

**Keywords:** Health policy, Statistics, Health services

## Abstract

The interest in applying machine learning in healthcare has grown rapidly in recent years. Most predictive algorithms requiring pathway implementations are evaluated using metrics focused on predictive performance, such as the *c* statistic. However, these metrics are of limited clinical value, for two reasons: (1) they do not account for the algorithm’s role within a provider workflow; and (2) they do not quantify the algorithm’s value in terms of patient outcomes and cost savings. We propose a model for simulating the selection of patients over time by a clinician using a machine learning algorithm, and quantifying the expected patient outcomes and cost savings. Using data on unplanned emergency department surgical readmissions, we show that factors such as the provider’s schedule and postoperative prediction timing can have major effects on the pathway cohort size and potential cost reductions from preventing hospital readmissions.

## Introduction

The world of clinical medicine has shown tremendous interest in artificial intelligence and machine learning^1^. Whereas machine learning algorithms are now commonplace in most other industries, these technologies have more recently been studied in medicine and have ignited a passion to find non-traditional ways to improve patient outcomes^2^. Although many of the same machine learning methodologies used in other domains are also used in healthcare, there are important differences in implementation^3^. In domains such as e-commerce, a machine learning algorithm is often used to make low-stakes decisions without human supervision^4^. For example, Google’s Adwords platform uses machine learning to predict the probability of a user clicking on an ad, which is then used to decide which ads to display in response to a user’s query^5^. Due to the high volume of queries and the fact that wrong decisions do not affect a person’s life, the Adwords platform directly makes such decisions, without human oversight.

In contrast, machine learning algorithms employed in healthcare typically involve predicting whether a patient will experience some type of adverse event^6–8^. Machine learning algorithms in healthcare are used to affect an individual’s disease course, which differs from how machine learning algorithms are used in other applications (such as online advertising) to target populations. The decisions that machine learning algorithms in healthcare inform are clinically important: an incorrect prediction of a patient’s risk can result in the patient receiving the wrong type of care or not receiving any care at all^9^. An algorithm’s predictions are thus typically reviewed by a clinician/provider and used to support effective decision making^10^. An effective algorithm or model will correctly anticipate patients who will experience an adverse event and allow time for a provider to potentially prevent the adverse event from occurring in the first place.

Thus, a critical factor in the deployment of any machine learning model in healthcare is the provider, who is guided by the algorithm’s predictions^11^. The presence of the provider introduces several important operational considerations that potentially complicate how the value of a machine learning algorithm is assessed, in terms of patient outcomes and the cost savings associated with mitigating the adverse event. These considerations include constrained access to providers, prediction timing relative to pathway resource availability, provider costs, the success rate of providers’ interventions, and the potential cost savings of the pathway interventions.

Typically, healthcare machine learning models are evaluated using traditional machine learning metrics. For example, receiver–operator characteristic curves and the area under the receiver–operator characteristic curve (AUROC; also known as the *c* statistic) are considered gold standards for quantifying predictive performance^12–14^. Other widely-used metrics include calibration-related metrics (such as the calibration slope and intercept)^15^, the net reclassification improvement^16^, and integrated discrimination improvement^16^. While these metrics and methods are valuable for understanding predictive performance, they lack clinical interpretability because they do not consider resource constraints, and do not consider the impact of the prediction made by the algorithm on the patient’s health. Specifically, they do not incorporate the providers’ schedules and the constraints on how many patients can be physically enrolled in a pathway; the timing of when the prediction becomes available to the providers; the cost of the providers; and the benefit of the providers both in terms of how many adverse events the providers can prevent, and the cost savings of this prevention. Although having merit, these commonly used performance metrics cannot be solely used to completely understand the overall benefit, both in terms of managing patient outcomes and managing costs, of a given machine learning model. Besides these predictively focused metrics, a notable body of recent work has approached predictive models from a decision-analytic perspective, and has proposed the metric of net benefit and the associated concept of a decision curve^17^. While these approaches help to compare different models, these approaches again do not account for resource constraints. The development of clinically applicable metrics and the integration of machine learning into clinical workflows have been identified as major challenges in the implementation of healthcare machine learning methods^18–21^, alongside other issues such as logistical difficulties in accessing data from a range of sources^18^, bias in training data^19^, whether model training should be site-specific^22^, and algorithmic interpretability^23^. In addition, recently proposed frameworks for translating machine learning into clinical practice have highlighted the demonstration of economic utility^20^ and rigorous evaluation of impact in terms of clinical outcomes and cost^21^ as important steps; such rigorous evaluation is likely to be of increasing importance in the future as machine learning-based software-as-a-medical-device (SaMD) systems become more regulated and standardized^24^.

In this paper, we propose a simulation model that allows clinicians and hospital leadership to assess the value of a machine learning model in a dynamic, provider-constrained setting. We specifically study the problem of using machine learning to predict postoperative 30-day hospital readmission via an emergency department, and compare previously developed predictive models that differ in predictive performance (as measured by AUROC) and in prediction timing (whether the prediction is available as early as the completion of surgery, or whether it is only available on the day of discharge). Our simulation model involves tracking the flow of patients as they complete surgery through discharge; calculating the risk of each patient using the predictive model; and then selecting the highest risk patients for intervention, subject to the provider’s availability and the pathway capacity (i.e., the maximum number of patients the provider can engage with on a given day). In this way, our simulation model aligns the predictions produced by a machine learning model with the provider.

At a high level, our simulation model is intended for clinicians and hospital leadership who are interested in implementing a machine learning model in real-time to guide the allocation of a constrained resource. Ideally, this simulation would be used early in the model development process, when one has already developed a candidate set of models, and is interested in forecasting the cost and prevention outcomes of each model in order to decide on the most suitable one. While we demonstrate our simulation model in the context of postoperative prediction of readmission for surgical patients, it applies to any other ML model that is used to allocate a constrained resource.

Our results highlight the complexity of using machine learning in clinical scenarios. In the context of readmissions, while better predictive performance translates to better impact in terms of the number of readmissions anticipated, this impact also highly depends on provider pathway enrollment and prediction timing to increase the patients’ window of availability. In addition, we show that the net cost savings also depend on these factors, as well as the provider cost and the effectiveness of the implemented pathway. Ultimately, our simulation model provides clinicians and administrators with a more clinically relevant assessment of the value of any predictive model in terms of patient outcomes and cost.

## Results

### Data extraction

Records for 19343 surgical admissions to the UCLA Ronald Reagan Medical Center (RRMC) over the period 2017–2018 were extracted. Of these, 12 admissions were excluded for being terminal organ donors. The remaining 19331 admissions were used as the input data to our simulation. Supplementary Table 1 summarizes the characteristics of this patient data set. Based on the extracted data, the simulation horizon was defined as a period of 847 days; we note that this period is slightly longer than two years, as some patients stay in the hospital past the end of 2018. For more information, we refer readers to the Methods section, which describes our data extraction process, our simulation, and our simulation-based performance metrics in greater detail.

To identify the patients satisfying our definition of a 30-day emergency department readmission, we first identified 1784 admissions that were followed by a subsequent 30-day emergency department visit. Of these, 36 were excluded due to the emergency department visit occurring on the same calendar day as discharge, and a further 779 were excluded for not resulting in a transfer to a subsequent non-emergency department location. This resulted in a set of 969 admissions (5.0% of the complete set of 19331 admissions) with readmission via the emergency department.

### Existing predictive and decision-analytic performance metrics

Table 1 displays the predictive and decision-analytic performance metrics for the four predictive models. This table has four sets of rows, for each of the four predictive models. In terms of metrics, it displays the AUROC; the discrete net reclassification improvement (NRI), measured relative to the HOSPITAL score and defined using the risk intervals [0,0.1), [0.1, 0.2), [0.2, 0.3), [0.3,1]; the continuous NRI, measured relative to the HOSPITAL score; the integrated discrimination improvement (IDI), measured relative to the HOSPITAL score; and the net benefit (NB), measured using a probability cutoff of 0.1. From the AUROC metric, we can see that HOSPITAL attains the lowest AUROC (0.7169), followed by the non-lab-based L1 logistic regression model, followed by LACE, and finally, the lab-based L1 logistic regression model, which attains an AUROC of 0.8541. The two NRI metrics and the IDI metric yield a similar ordering of the models: the two L1 logistic regression models and the LACE model all achieve an improvement over the HOSPITAL model in reclassification and discrimination, with the lab-based L1 logistic regression model achieving the highest improvement, and the non-lab-based L1 logistic regression model achieving the smallest improvement. With regard to the NB metric, we can see that at a probability cutoff of 0.10, the HOSPITAL, LACE, and non-lab-based L1 logistic regression models achieve similar net benefits of 0.49%, 0.50%, and 0.53%, respectively, and the lab-based L1 logistic regression model achieves the highest net benefit of 1.8%. This ordering of the models is again qualitatively similar to that obtained from AUROC.

**Table 1:** Predictive metrics for the four models.

Model	AUROC	NRI	NRI	IDI	NB
		(discrete)	(continuous)		
HOSPITAL	0.7169	–	–	–	0.4886
	(0.7017, 0.7325)	–	–	–	(0.2960, 0.6627)
LACE	0.7367	0.1403	0.3996	0.0218	0.4978
	(0.7212, 0.7512)	(0.1014, 0.1793)	(0.338, 0.4613)	(0.0181, 0.0256)	(0.2626, 0.7232)
L1LR	0.7280	0.0523	0.3560	0.0201	0.5299
(no labs)	(0.7108, 0.7431)	(0.0139, 0.0907)	(0.2953, 0.4239)	(0.0157, 0.0245)	(0.3442, 0.7219)
L1LR	0.8541	0.4171	0.9721	0.1263	1.8082
(with labs)	(0.8442, 0.8656)	(0.3740, 0.4602)	(0.9128, 1.0314)	(0.1157, 0.1368)	(1.5507, 2.0692)

NRI (discrete), NRI (continuous), and IDI are calculated relative to HOSPITAL. NRI (discrete) is based on the risk intervals [0,0.1), [0.1,0.2), [0.2, 0.3), [0.3, 1]. NB is measured at a probability cutoff of $\bar p$ = 0.1 and is given as a percentage. The 95% bootstrap confidence interval (based on 1000 bootstrap simulations) is given underneath each metric. The results for the lab-based L1LR model correspond to the model from Mišić et al.^28^ at a cutoff of 1 day after the completion of surgery. The NRI and IDI metrics were computed using the PredictABEL package in R^41^.

### Simulation-based patient performance metrics

Table 2 displays the simulation-based patient performance metrics for the four predictive models over the simulation horizon (see the “Performance metrics” section under the “Methods” section for a precise definition of each of the metrics). This table shows three different provider schedules: in the first, the provider sees 8 patients once a week every Monday; in the second, the provider sees 8 patients on Monday and 8 on Wednesday; and in the third, the provider sees 8 patients every day from Monday to Friday. The patients seen (PS) metric measures how many patients the provider sees over the simulation horizon. For example, when the provider sees 8 patients every Monday, the four different predictive models result in the provider seeing between 845 patients (for HOSPITAL and LACE) and 866 patients (for L1 regularized logistic regression with the lab-based features). As the provider’s schedule includes more days, the PS metric increases. Evaluating the Monday–Wednesday schedule, the number of patients seen ranges from 1688 to 1705, whereas with the Monday-to-Friday schedule 4196–4215 patients are seen. In general, HOSPITAL and LACE result in a smaller number of patients seen, because these two predictive models can only be applied on the discharge date. For example, if the provider only sees patients on Mondays and a patient is discharged on a Tuesday, that patient will not be selected by a provider using HOSPITAL or LACE.

**Table 2:** Simulation-based patient performance metrics for the four models.

Schedule	Capacity	Method	PS	RA	ERP
M	8	L1LR (with labs)	866	258	25.8
			(858, 874)	(228, 281)	(22.8, 28.1)
M	8	L1LR (no labs)	861	155	15.5
			(855, 869)	(133, 176)	(13.3, 17.6)
M	8	LACE	845	91	9.1
			(832, 849)	(67, 101)	(6.7, 10.1)
M	8	HOSPITAL	845	86	8.6
			(832, 849)	(67, 101)	(6.7, 10.1)
M W	8	L1LR (with labs)	1705	423	42.3
			(1696, 1713)	(381, 454)	(38.1, 45.4)
M W	8	L1LR (no labs)	1699	263	26.3
			(1691, 1707)	(229, 289)	(22.9, 28.9)
M W	8	LACE	1688	178	17.8
			(1673, 1695)	(150, 198)	(15, 19.8)
M W	8	HOSPITAL	1688	173	17.3
			(1673, 1695)	(142, 190)	(14.2, 19.0)
M T W R F	8	L1LR (with labs)	4199	672	67.2
			(4189, 4213)	(629, 720)	(62.9, 72)
M T W R F	8	L1LR (no labs)	4196	502	50.2
			(4186, 4207)	(461, 548)	(46.1, 54.8)
M T W R F	8	LACE	4215	456	45.6
			(4190, 4222)	(405, 486)	(40.5, 48.6)
M T W R F	8	HOSPITAL	4215	437	43.7
			(4190, 4222)	(375, 450)	(37.5, 45.0)

Under “Method”, “L1LR” denotes L1 regularized logistic regression. “Schedule” indicates the days of the week on which the provider works (M = Monday, T = Tuesday, W = Wednesday, R = Thursday, F = Friday). For the last four columns, PS denotes the number of patients seen (how many patients were seen by the provider over the simulation horizon); RA denotes the number of readmissions anticipated (how many patients were seen by the provider and had an ER readmission); and ERP denotes the expected readmissions prevented (RA multiplied by the effectiveness coefficient), assumed to be 10%. The 95% bootstrap confidence interval (based on 1000 bootstrap simulations) is shown underneath each metric.

The readmissions anticipated (RA) metric measures how many of those patients seen by the provider have unplanned readmission. When the provider sees patients on Mondays and uses the L1 regularized logistic regression model without the lab-based features, the provider correctly anticipates 155 readmissions. When the provider uses the L1 logistic regression model with the lab-based features and follows the same schedule, the provider correctly anticipates 258 readmissions; relative to the non-lab-based model, this constitutes an improvement of 66%. Using HOSPITAL and LACE only results in 86 and 91 readmissions, respectively, being correctly anticipated with the same Monday-only schedule; relative to HOSPITAL and LACE, the lab-based L1 logistic regression model results in an improvement of over 183%.

The difference between the lab-based and the non-lab-based model is driven by the difference in their predictive ability. As shown in Table 1, the AUROC for the lab-based model on this data set is 0.8541, whereas it is only 0.7280 for the non-lab-based model. A model with a perfect AUROC of 1 would ensure that the number of readmissions anticipated by the provider is maximized, subject to the constraint imposed by the provider’s schedule.

The difference between the non-lab-based model versus HOSPITAL and LACE, however, is not due to predictive ability, because the non-lab-based model, HOSPITAL, and LACE all achieve AUROCs in the range 0.71–0.74. The difference arises because of the schedule and the availability window. With HOSPITAL and LACE, the provider is restricted to only seeing patients that are being discharged on a pathway enrollment day. Thus, while there may be high-risk postoperative patients that are available and that could in theory be seen, the provider must select from among those that are being discharged, and the set of patients selected by the provider will include patients that are low-risk. In contrast, with the non-lab-based model, the provider can see a patient on any day from the completion of surgery to discharge. Thus, on any given Monday, the provider can select patients who recently completed surgery and are high-risk, as well as high-risk patients who have already been in the hospital for some time and have not yet been seen.

As discussed above, under a Monday-only schedule, the lab-based model improves on the readmissions anticipated over the non-lab-based model, HOSPITAL and LACE. When the provider sees patients on Mondays and Wednesdays, this benefit decreases: the lab-based model improves on the readmissions anticipated by about 60% over the non-lab-based model (423 vs. 263), and by about 138% over HOSPITAL and LACE (423 vs. 173 and 178). When the provider sees patients on Monday through Friday, this benefit further decreases to about 34% relative to the non-lab-based model (672 vs. 502), about 54% relative to HOSPITAL (672 vs. 437) and about 47% relative to LACE (672 vs. 456). The reason that this benefit decreases is because with a larger schedule, the provider can enroll more patients and is less constrained; stated differently, any deficiencies in a model’s predictive ability can be compensated for by seeing more patients. Taken to the extreme, if providers could theoretically enroll all of the surgical patients without provider cost concerns, then all four predictive models would benefit patients identically.

The improvements in the readmissions anticipated by the L1 logistic regression models directly translate into improvements in the ERP metric, which measures the expected number of readmissions prevented. For example, assuming an effectiveness constant of 10% and the provider seeing up to 8 patients every Monday, the lab-based L1 logistic regression model is expected to prevent 26 readmissions, whereas HOSPITAL and LACE are expected to prevent only 9.

### Simulation-based cost performance metrics

Table 3 reports on the cost performance metrics for the different predictive models under the same three provider schedules as in Table 2. (The precise definition of each metric is provided under the “Performance metrics” subsection of the “Methods” section.)

**Table 3:** Simulation-based cost performance metrics for the four different models.

Schedule	Capacity	Method	ERC	PC	ERCS	ENCS
M	8	L1LR (with labs)	3,745,900	72,600	374,590	301,990
			(3310498, 4078118)		(331050, 407812)	(258450, 335212)
M	8	L1LR (no labs)	2,232,000	72,600	223,200	150,600
			(1914840, 2534760)		(191484, 253476)	(118884, 180876)
M	8	LACE	1,320,900	72,600	132,090	59,490
			(1015000, 1540878)		(101500, 154088)	(28900, 81488)
M	8	HOSPITAL	1,252,400	72,600	125,240	52,640
			(975290, 1471510)		(97529, 147151)	(24929, 74551)
M W	8	L1LR (with labs)	6,142,700	145,200	614,270	469,070
			(5528685, 6579343)		(552869, 657934)	(407669, 512734)
M W	8	L1LR (no labs)	3,790,700	145,200	379,070	233,870
			(3297600, 4167420)		(329760, 416742)	(184560, 271542)
M W	8	LACE	2,577,200	145,200	257,720	112,520
			(2170413, 2865298)		(217041, 286530)	(71841, 141330)
M W	8	HOSPITAL	2,513,300	145,200	251,330	106,130
			(2058753, 2762815)		(205875, 276282)	(60675, 131082)
M T W R F	8	L1LR (with labs)	9,776,000	453,750	977,600	523,850
			(9153885, 10474863)		(915389, 1047486)	(461639, 593736)
M T W R F	8	L1LR (no labs)	7,265,500	453,750	726,550	272,800
			(6666153, 7921670)		(666615, 792167)	(212865, 338417)
M T W R F	8	LACE	6,618,400	453,750	661,840	208,090
			(5861268, 7052718)		(586127, 705272)	(132377, 251522)
M T W R F	8	HOSPITAL	6,350,800	453,750	635,080	181,330
			(5441198, 6520680)		(544120, 652068)	(90370, 198318)

Under “Method”, “L1LR” denotes L1 regularized logistic regression. “Schedule” indicates the days of the week on which the provider works (M = Monday, T = Tuesday, W = Wednesday, R = Thursday, F = Friday). For the last four columns, ERC denotes the expected readmission cost in dollars (the sum of the expected readmission cost, according to HCUP data, for those patients seen and who had an ER readmission); PC denotes the provider cost in dollars (the cost of giving the provider).

**Fig. 1:**
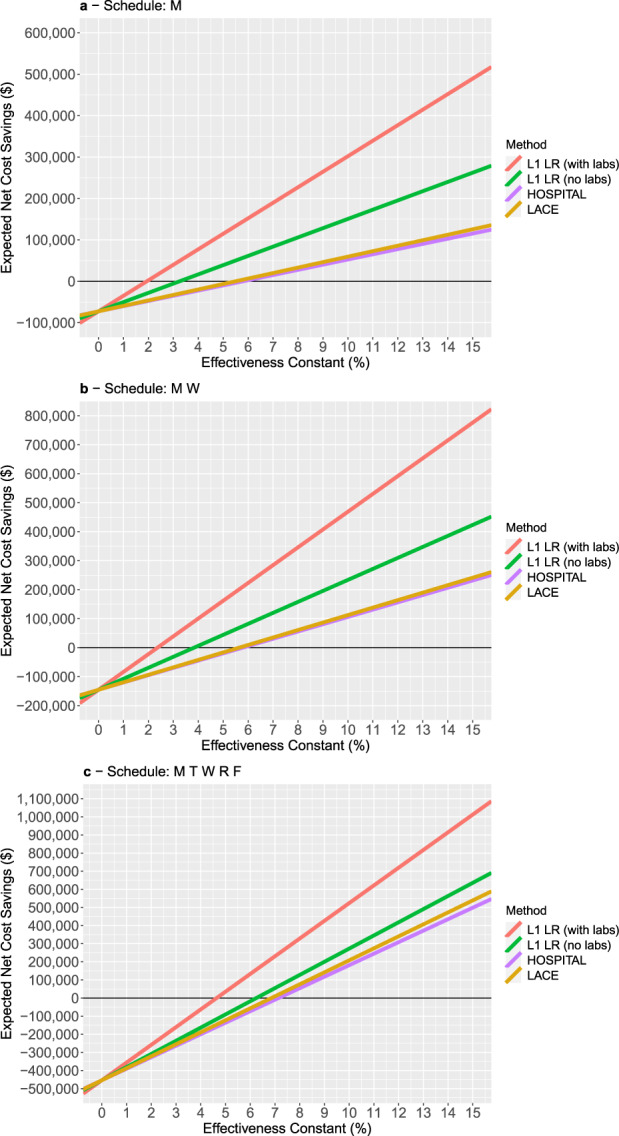
Expected net cost savings as a function of the effectiveness constant, for the three different schedules. Under “Method”, “L1LR” denotes L1 regularized logistic regression. “Schedule” indicates the days of the week on which the provider works (M = Monday, T = Tuesday, W = Wednesday, R = Thursday, F = Friday). Panel **a** corresponds to a Monday-only schedule, panel **b** corresponds to a Monday/Wednesday schedule, and panel **c** corresponds to a Monday - Friday schedule.

For the Monday-only schedule, the lab-based L1 logistic regression model selects a set of patients over the simulation horizon whose expected readmission cost is approximately $3.7 million. This is larger than the readmission cost of the patients selected by the non-lab-based L1 logistic regression model ($2.2 million), as well as HOSPITAL and LACE ($1.3 and $1.3 million, respectively). As in Table 2, the difference between the lab-based and non-lab-based L1 logistic regression models is driven by the difference in their predictive ability, while the difference between the non-lab-based L1 logistic regression model and the two scoring rules (HOSPITAL and LACE) is driven by the difference in the availability window. The provider cost for this schedule is $72,600, which we note is the same across all four models. Under the assumption of an effectiveness constant of 10%, the expected readmission cost savings for the four models ranges from roughly $380,000 for the lab-based L1 logistic regression model to roughly $125,000 and $132,000 for HOSPITAL and LACE, respectively. The expected net cost savings, which is the difference of the expected cost savings and the provider cost, ranges from roughly $302,000 for the lab-based L1 logistic regression model, to roughly $50,000–$60,000 for HOSPITAL and LACE. We emphasize that these results are based on the assumption of an effectiveness constant of 10%; under different values of the effectiveness constant, these expected net cost savings can change drastically (as we will subsequently show; cf. Fig. 1). Notwithstanding this assumption, these results illustrate how differences in both predictive ability and the availability window of a model can directly translate into differences in expected cost and financial performance.

When we consider the other schedules—Monday/Wednesday and Monday-to-Friday—we observe that as the schedule enlarges, the expected readmission cost and the expected readmission cost savings increase, because the provider sees more patients and anticipates more readmissions. The provider cost also increases, as the provider works a larger number of hours. The relative difference in expected net cost savings between the lab-based L1 logistic regression model and HOSPITAL/LACE decreases as the provider’s schedule enlarges. This mirrors our observations with the RA metric in Table 2, where we saw that seeing more patients leads to a reduced performance gap between the methods.

Figure 1 plots the expected net cost savings as a function of the effectiveness constant for the four predictive models, for each of the three schedules. These plots suggest that if the effectiveness constant is sufficiently low, then it is not cost-effective to use the provider. The plots also allow us to infer the break-even effectiveness constant for each of the models. For example, for the Monday-only schedule, the lab-based L1 logistic regression model leads to positive net cost savings when the effectiveness constant is roughly 2% or higher; in contrast, for HOSPITAL and LACE, one needs the effectiveness constant to be >5.5–6% for the net cost savings to become positive. These plots also suggest that the break-even effectiveness constant generally increases as the provider schedule is enlarged. This implies that for pathways with a low effectiveness constant, one should consider a smaller provider schedule to stay profitable; conversely, a larger provider schedule is only justified when the pathway has a higher effectiveness constant.

### Additional results

In the Supplementary Information, we provide patient and cost performance results for alternate values of 5 and 20% for the effectiveness constant, with the same provider cost model (Supplementary Tables 2 and 3). In Supplementary Table 4, we also report cost results under two different provider cost scenarios, where we change the provider cost by factors of 0.5 and 2.0 (provider cost is half and twice the base case in Table 3, respectively). Lastly, in Supplementary Table 5, we consider an alternate provider cost structure, where the provider cost is based on a cost per patient selected (one of $100, $200, or $300).

## Discussion

Our simulation model yields three important insights. The first is that improvements in the predictive performance of a readmission prediction model directly translate to more effective allocations of limited readmission prevention resources. This is borne out in the comparison between the lab-based and non-lab-based L1 regularized logistic regression model, which have the same availability window and attain AUROCs of 0.8541 and 0.7280, respectively. Both models result in the provider seeing roughly the same number of patients in each schedule, but the lab-based model anticipates a much larger number of readmissions, and results in significantly higher expected net cost savings. Our simulation model allows clinicians and hospital leadership to directly see how differences in AUROC will translate into the added readmissions that a prevention pathway correctly anticipates, and the increased cost savings from correctly anticipating those additional readmissions.

The second insight is that the timing of a prediction, as captured through the patients’ availability window, also impacts the ultimate efficacy of how providers can enroll patients into a prevention pathway. In our results, the non-lab-based regularized logistic regression model, HOSPITAL and LACE all achieve comparable AUROCs, but HOSPITAL and LACE anticipate a much smaller number of readmissions, because those two models can only be applied to a patient on the day of discharge, while the non-lab-based regularized logistic regression model can be applied as soon as the patient completes the surgery. This directly translates to lower expected net cost savings. While it is intuitive that having access to a prediction earlier is better than later, the value of our simulation model is in quantifying the impact of this variable in terms of patient care (how many readmissions are anticipated) and cost (expected net cost savings).

The third insight is that resource constraints and costs also impact model efficacy. Our results show that the improvement in the number of readmissions anticipated by the two machine learning models over HOSPITAL and LACE is highest for the most constrained schedule, and decreases as the provider’s schedule is expanded to enroll a larger number of patients. Thus, in quantifying the value of improved predictive performance, our results suggest that one must account for the capacity of resources that will be directed by a predictive model. Through our simulation model, a clinician that is deciding how to best deploy a given machine learning model can evaluate multiple schedules and choose the schedule that leads to the most attractive performance in terms of provider cost, readmissions anticipated, and expected net cost savings.

Taken together, these three insights suggest that while standard metrics used in healthcare machine learning (such as AUROC) are helpful for characterizing predictive performance, these metrics do not fully quantify the value of models in potentially improving patient outcomes and reducing costs. In particular, a model’s AUROC value does not capture any element of when the model’s predictions become available; it does not account for the schedule of the provider that will use the model to direct, and it does not account for the cost associated with the provider and the expected savings associated with each readmission. As a stark example of the importance of costs and savings, our sensitivity analyses suggest that if the effectiveness decreased to 5% (as in Supplementary Table 3) or if the provider cost were twice as high (Supplementary Table 4), the expected net cost savings generated by HOSPITAL and LACE in all three schedules are negative; under such scenarios, our simulation model would suggest that there is no financial justification to using HOSPITAL and LACE to guide provider allocation. This type of insight is absent from solely predictive metrics like AUROC, but is of critical importance for implementation.

Ultimately, predictive models exist to enable decision makers to judiciously allocate limited resources over time so as to enroll patients in care pathways, improve quality of care, and reduce costs. Although we have focused on readmission prediction, resource constraints and limited intervention time windows are essential features of many other risk prediction problems that arise in healthcare when rolling out clinical pathways. Our results highlight the importance of simulating any predictive model under consideration in a simulation model that accounts for the temporal dynamics of patient flow, as well as the constraints that limit when and to what extent resources can be used to affect patients. Our simulation model can thus be viewed as a stepping stone from initial machine learning model development—which involves defining features, model estimation, and out-of-sample testing—to the real-time implementation of the predictive model within an electronic health record (EHR) system. More importantly, it can be used as a guide for providing financial justification when proposing AI-backed clinical pathways to hospital leadership.

There are several limitations to our analysis. First, we assume that if a provider sees a patient that would eventually get readmitted, that the provider can prevent the readmission of that patient with some probability, which is given by the effectiveness constant. While there is much data on techniques to prevent hospital readmission, we are in the process of creating a sound pathway with nothing yet trialed on patients. We do not make assumptions on the type of pathway; we only assume that the pathway’s effectiveness constant is uniformly 10% across all patients (and provide additional results in our Supplementary Information for alternate values of 5 and 20%). We note that the recent paper of Leppin et al.^25^ presents a meta-analysis of studies on readmission prevention and suggests that the average reduction in readmissions is 18%. Although this number is an average over studies that have considered different populations and different types of interventions, this would suggest that our results, which are based on a lower effectiveness constant value of 10%, are a conservative estimate of the efficacy of the different models. It is straightforward to adjust the simulation model details (such as the provider capacity and the intervention effectiveness) to more accurately characterize a machine learning model’s performance. In addition, it is also straightforward to modify the simulation model to incorporate a patient-specific effectiveness constant that would depend on the characteristics of the patient and their index admission.

Second, our analysis makes assumptions on the costs of readmissions and the cost of setting up the provider pathway. The readmission costs are derived from Healthcare Cost and Utilization Project (HCUP) data, which represent national averages and do not solely focus on surgical patients; the actual costs at a given institution could differ significantly. Given more detailed and higher fidelity data on the cost of each readmission, it is again straightforward to modify the simulation model to derive more precise estimates of net cost savings. With regard to the cost of the prevention pathway, we note that it is also easy to modify the simulation model to accommodate more complex cost structures. For example, in the Supplementary Information, we consider an alternate cost structure where the cost is a variable cost that scales linearly in the number of patients enrolled in the intervention pathway. Our analysis also does not include the cost of acquiring the data and setting up the algorithm for real-time use. Although developing the data warehousing capability to support a real-time algorithm would be an additional upfront cost, such an investment could potentially be amortized over the development of many other clinical decision support tools.

Third, our simulation model does not address logistical issues in the implementation of the machine learning model, and in particular, it does not consider how the machine learning model will fit with the existing clinical workflow. As noted previously, the application of our simulation model to the context of readmission prevention assumes that there is a dedicated provider guided by a machine learning model who is able to see a certain number of patients on certain days of the week. This workflow does not currently exist at UCLA RRMC, and we are not aware of such a similar workflow elsewhere. Thus, it is difficult to foresee potential issues with relaying a predictive model’s predictions to a provider at this point in time. Studying how the predictions from the machine learning model should be relayed to the provider (e.g., when the provider should be given those predictions, and how much auxiliary information the provider should be given), is an important direction for future research. Our simulation model also does not account for the nature of the machine learning model in terms of issues like interpretability, trust, bias, and confounding. While these issues are typically addressed in the model development stage, they are important to consider because they can affect the adherence of providers to the machine learning model’s recommendations, and the potential information that the provider can glean from the model. For example, if the predictions are produced by an interpretable model, the provider can see how the prediction is made for a given patient and can potentially obtain insight into the underlying causes of the patient’s increased risk of readmission, which can help the provider with applying the intervention.

Lastly, we note that our simulation model compares different predictive models under different provider schedules in terms of the number of readmissions prevented and in terms of the net cost savings of those readmissions prevented. Another important dimension to consider is the effect of the provider on the patients’ health, in terms of clinical outcomes and the patient’s quality of life. This raises a concern that the provider could make changes in a patient’s care that would negatively impact the outcome and cause a readmission that was not likely prior to the patient engagement. This dimension of considering the patient’s health is currently not captured in our simulation model; including it constitutes an important direction of future research.

In conclusion, we proposed a new simulation model to demonstrate the rich clinical and administrative value of machine learning models that is not captured by gold standard metrics for predictive performance, such as AUROC. As this work evolves, we hope to transform this into a true patient trial to compare our simulated in-silico results against real in-vivo outcomes.

## Methods

### Data extraction

This study (UCLA IRB #18-000630) qualified for UCLA IRB exception status (waiver of consent) due to having no direct contact with patients and using de-identified data. We used the Perioperative Data Warehouse developed by the UCLA Department of Anesthesiology and Perioperative Medicine, which has been described in detail in other references^26,27^.

We extracted de-identified data corresponding to all surgical patients that were admitted at the UCLA Ronald Reagan Medical Center (RRMC) in 2017 and 2018 and underwent a procedure with anesthesia. We extracted de-identified data on the date of admission and discharge of each patient, so as to maintain the exact sequence of patient admissions and discharges, and to be able to realistically simulate the providers’ workflow. We defined the start date of the simulation as the earliest admission date over the extracted patients, and the end date as the latest discharge date over the extracted patients.

For each admission, we define a binary variable to indicate whether the patient had an emergency department readmission. We follow the prior work of Mišić et al.^28^ in defining an emergency department readmission as an event when a patient enters the hospital through the emergency department within 30 days of their first surgical discharge and is subsequently moved to a non-emergency department inpatient location. An exception was made for patients that were discharged and admitted on the same calendar day to better align with the Centers for Medicaid and Medicare Services (CMS) definition^29^. We additionally make this exception as the same definition is used in Mišić et al.^28^, allowing us to directly connect our simulation model to the results of this previous work, and reuse the models from this prior work.

### Simulation model

The goal of our simulation model was to accurately evaluate how an unplanned readmission prediction model would allocate a constrained provider to patients. We implemented and evaluated our simulation model in the R and Julia programming languages^30,31^.

#### Predictive models

The primary input to our simulation model was a predictive model, which takes as input the patient’s data at a given point in time relative to their admission and outputs a predicted probability of future ED-based readmission. We considered four different types of predictive models, which we briefly describe below. The first two models are the lab-based and non-lab-based L1 regularized logistic regression models from Mišić et al.^28^. These models were developed using a distinct subset of admissions from 2013 to 2016, and were not trained using any of the data extracted for the purpose of this simulation. We use the terms lab-based/non-lab-based to indicate whether the model uses the lab-based features described in Mišić et al.^28^ as predictor variables. The other two models that we considered are the HOSPITAL score^32^ and the LACE score^33^. We refer readers to the aforementioned references^28,32,33^ for more information on the variables used in these four models.

#### Model availability

Each model has an associated availability window, which is a window of time during which the prediction from the model is available, and the patient may be selected for intervention. For the regularized logistic regression models, the availability window was defined as the period from the day on which surgery is completed to the day of discharge. For the lab-based model, the lab-based features are defined using all lab measurements that have occurred up to a given point in time during surgery; thus, the prediction from the lab-based regularized logistic regression model can change with each day in the availability window. We updated the prediction after day 1 (the day of surgery completion) and day 2 (one day after surgery completion) of the availability window. We did not update the prediction after day 2 of the availability window, as it was shown in Mišić et al.^28^ that the AUROC performance of the model plateaus at 1.5–2 days after the completion of surgery.

An essential variable required by both HOSPITAL and LACE is the patient’s length of stay, which will typically not be known upon the completion of surgery. Thus, we assumed that the availability window for HOSPITAL and LACE would consist of only a single day, which is the day of discharge.

#### Simulated provider

We assumed a single provider following a weekly schedule that defines a set of days on which the provider is able to see patients. On each day in the schedule, the provider was assumed to have a limit on the number of patients that can be seen, which we refer to as the daily capacity. For example, a Monday and Wednesday schedule with a daily capacity of 8 patients means that the provider can see up to 8 distinct patients on Monday and 8 on Wednesday. The provider cannot see a patient that is in the hospital on a given day if that day does not fall in the patient’s availability window.

**Fig. 2:**
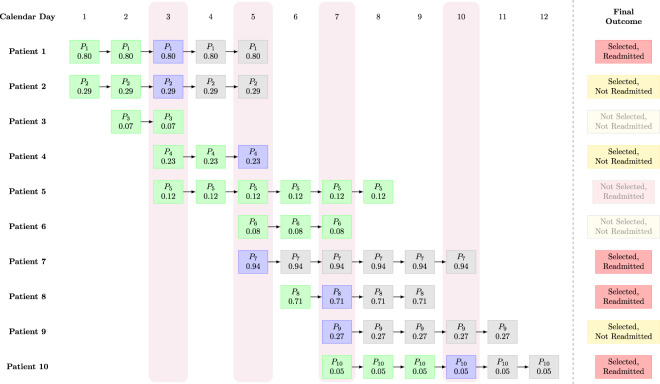
Visualization of an example of the provider workflow in the simulation. Each square cell is a postoperative patient in the hospital on a particular day. Within each cell, *P*_*i*_ indicates the *i*th patient, while the number underneath indicates the predicted readmission probability on that day (e.g., for patient 1 on day 1, this is 0.80). If there is no square cell, then the patient has either not yet completed surgery, or has been discharged. For example, patient 5 completes surgery on day 3 and is discharged on day 8; before day 3, the patient is either still in surgery or has not yet been admitted; after day 8, the patient is no longer present in the hospital. The vertical rectangles shaded in light purple indicate days on which the provider selects patients. In this example, we assume that the provider works on days 3, 5, 7, and 10, and has a capacity of two patients, i.e., the provider can select at most two patients to apply the intervention to on each of those four days. On a given day, if a patient is available for selection and has not yet been selected, they are shaded in green. On a given day, if the provider selects them, they are shaded in blue. After the provider selects them and applies the intervention pathway, the patient is shaded in gray for the remaining days of their hospital stay. As an example of the provider selection process, observe that on day 3, there are 5 postoperative patients available (patients 1, 2, 3, 4, and 5). The provider selects patients 1 and 2, because these two patients have the highest predicted probability of readmission (0.80 and 0.29, respectively). After patients 1 and 2 have been selected, they cannot be selected again; for the remaining days that those two patients are in the hospital, they are indicated with the gray square cells. Notice that patient 4, who was not selected on day 3, is later selected by the provider on day 5; in this case, patient 4 was not among the top two patients on day 3 but became one of the top two patients on day 5. On the other hand, patient 3 is discharged before day 5; thus, this patient is lost and never ends up receiving the intervention pathway. We also note that on day 10, the provider only selects one patient (patient 10), because the other two patients (patients 7 and 9) were already selected previously (days 5 and 7, respectively). The rectangular cells on the right summarize the final outcome with each patient, with regard to whether or not they were selected by the provider, and whether or not they eventually experienced an ED readmission. In this example, there are a total of 7 patients who are selected, so the patients seen (PS) metric is 7. The number of patients selected and who get readmitted is exactly 4, so the RA metric is 4. Assuming an effectiveness constant of 10%, the ERP metric is 4 × 10% = 0.40.

We simulated the provider as follows. We started the simulation on the first day in the simulation horizon that falls in the provider’s schedule. We looked up the set of patients such that the current day falls within the patient’s availability window. We removed any patients that have already been seen by the provider on any previous day. For the remaining patients, we used the predictive model to compute the predicted probability of readmission. We sort those patients in decreasing order of the predicted readmission probability. We assumed that the provider applies the prevention pathway to the patients in that order, up to the limit on the number of patients defined by the provider’s daily capacity. For example, if 10 patients are available and the provider’s daily capacity is 3, the provider will select the top 3 patients in predicted readmission probability. We then moved on to the next day in the simulation horizon that falls in the provider’s schedule, and repeated the procedure until reaching the last day in the simulation horizon. Figure 2 provides a visualization of a small example of the simulated workflow.

### Provider effectiveness

We assumed that the prevention pathway applied by the provider is imperfect. We defined the effectiveness constant as a number between 0 and 100% that determines what fraction of eventual readmissions are successfully mitigated. For example, if the provider selects 50 patients of which 5 are eventually readmitted, and the effectiveness constant is 20%, then the provider is expected to prevent exactly 1 (20% of 5 readmissions). We considered a conservative value of the effectiveness constant of 10%, which is uniform across all index admission types, although in the results we considered alternate values of 5 and 20% as well.

### Performance metrics

We defined several simulation-based patient performance metrics. We defined the patients seen (PS) as the number of patients selected by the provider for the prevention pathway. We defined the readmissions anticipated (RA) to be the number of patients that were selected by the provider and that resulted in an emergency department-based hospital readmission. We defined the expected readmissions prevented (ERP) as RA multiplied by the effectiveness constant.

We defined several simulation-based metrics quantifying the provider’s cost impact. We defined the readmission cost (RC) to be the total expected cost of those patients selected by the provider that resulted in an ER readmission. To compute this, we used data from the Healthcare Cost and Utilization Project (HCUP), which provides national average readmission costs for different diagnosis groups, reported in Bailey et al.^34^. For a given patient, we looked up their ICD10 code in Table 3 of Bailey et al., and used the associated average readmission cost (Supplemental Table 2 of Bailey et al.) as the cost of that patient’s readmission. In the case that the patient had multiple ICD10 codes, we used the highest matching average readmission cost, and in the case that none of the ICD10 codes matched, we defaulted to the average readmission cost. We defined the expected readmission cost savings (ERCS) to be the readmission cost multiplied by the effectiveness constant, which represents the cost savings from applying the prevention pathway to the selected patients.

We also defined the provider cost (PC) as the total compensation paid to the provider over the simulation horizon. We assumed that the provider is compensated at a rate of $75 per hour, and each patient seen by the provider requires an hour of time; thus, the weekly compensation can be determined from the provider’s schedule and daily capacity. For example, a Monday and Wednesday weekly schedule and a daily capacity of 8 patients would translate to weekly hours of 16 and a weekly compensation of $75 × 16 = $1200. If the provider’s hours exceed 20 h a week, the weekly compensation is further increased by 25%, to account for the difference between part-time providers and full-time providers who would receive additional compensation in the form of benefits. Our assumptions here were motivated by a review of salaries for nurse practitioners at the UCLA RRMC. There are some costs that are not accounted for by physician oversight, but these could be added to the model in the form of a baseline cost that is not associated with hourly provider utilization.

Lastly, we defined the expected net cost savings (ENCS) as the difference of the expected readmission cost savings and the provider cost. This metric was intended to quantify the benefit of using a model for patient selection, net of the cost of implementing the model through the provider.

### Comparison to existing predictive and decision-analytic metrics

We briefly review existing metrics for quantifying predictive and decision-analytic performance that have been proposed in the healthcare machine learning literature, and contrast them to our proposed simulation-based metrics.

#### Area under the receiver–operator characteristic curve (AUROC)

The AUROC metric, also known as the *c* statistic, is mathematically defined as

1 $${\mathrm{AUROC}} = \frac{1}{{N_ + N_ - }}\mathop {\sum }\limits_{i \in S_ + } \mathop {\sum }\limits_{j \in S_ - } 1\left( {\hat Y_i \,>\, \hat Y_j} \right)$$

where $\hat Y_i$ is the predicted probability of readmission for patient *i*, $S_ +$ is the set of patients that are readmitted, $S_ -$ is the set of patients that are not readmitted, $N_ +$ is the number of patients that are readmitted, $N_ -$ is the number of patients that are not readmitted, and $1(\hat Y_i \,>\, \hat Y_j)$ is 1 if $\hat Y_i \,>\, \hat Y_j$ and 0 otherwise. The AUROC metric measures the discrimination ability of a predictive model. It has the following interpretation: given a random patient that goes on to be readmitted and a random patient who does not, the AUROC measures the probability that the predictive model correctly distinguishes between the two patients, i.e., that it assigns a higher predicted probability to the patient who gets readmitted than the one who does not. The AUROC metric ranges from 0.5 to 1. A value of 1 corresponds to perfect discrimination; in other words, a patient that will be readmitted is always assigned a higher risk score than one that will not be readmitted. On the other hand, a value of 0.5 corresponds to discrimination that is “as good as a random”; stated differently, given a patient that is readmitted and one that is not, our model is no better than if we were to pick the readmitted one at random from those two patients (which would result in being correct 50% of the time).

#### Calibration plot, slope, and intercept

The AUROC metric measures the discrimination ability of a model, which is its tendency to give higher risk scores to patients that get readmitted than those who do not get readmitted. Besides discrimination, another dimension of predictive performance is calibration, which is how well does the predicted probability of readmission correspond to the actual probability of readmission. Calibration is typically assessed using a calibration plot, which is constructed as follows. One computes the predicted probability of readmission for each patient, and buckets the patients by their predicted probabilities according to the deciles of their predicted probabilities. For each of the deciles $i = 1, \ldots ,10$, one computes the average predicted probability of readmission $Q_i$, and the empirical probability $\hat Q_i$. One then plots the pairs $\left( {Q_1,\hat Q_1} \right), \ldots ,(Q_{10},\hat Q_{10})$ on a scatter plot. Ideally, $Q_i$ should be close to $\hat Q_i$, and so the resulting calibration curve should be as close as possible to the *y* = *x* line (corresponding to a 45 degree line through the origin).

In addition to the calibration plot, one can compute two calibration metrics, which are the calibration slope and intercept^15^. Given predicted probabilities for each data point $\hat Y_i$, one assumes that the log-odds of the actual binary outcomes are a linear function of the log-odds of the predicted probabilities produced by the model, i.e.,

2 $$\log \frac{{p_i}}{{1 - p_i}} = \alpha + \beta \log \frac{{\hat Y_i}}{{1 - \hat Y_i}}$$

where $p_i$ is the actual probability for observation *i*, *α* is the calibration intercept and *β* is the calibration slope. The values of *α* and *β* can be estimated using logistic regression. Ideally, *α* should be as close as possible to zero, and *β* as close as possible to 1. A value of *α* that is below 0 indicates that the predictive model systematically overestimates the true probability of readmission, while a value above 0 indicates that the predictive model systematically underestimates the true probability^15^. Similarly, a value of *β* that is greater than 1 indicates that the predicted probabilities do not vary sufficiently; a value of *β* between 0 and 1 suggests that there is too much variation in the predicted probabilities, and a value of *β* below 0 suggests that the predicted probabilities vary in the wrong direction (low probabilities are predicted for observations for which the actual probability is high, and vice versa)^15^.

#### Integrated discrimination improvement (IDI)

The IDI metric is a measure of the improvement in discrimination of one model over another model^16^. The two models could be from the same model class but vary in their predictors, or they could be from different model classes. To define it formally, we first define the discrimination slope of a model as

3 $${\mathrm{DS}} = \frac{{\mathop {\sum }\nolimits_{i \in S_ + } \hat Y_i}}{{N_ + }} - \frac{{\mathop {\sum }\nolimits_{i \in S_ - } \hat Y_i}}{{N_ - }}$$

In words, the discrimination slope is the difference in the average predicted probability between patients that are readmitted and patients that are not. Given two models $m_1$ and $m_2$, the IDI is defined as

4 $${\mathrm{IDI}}_{m_1,m_2} = {\mathrm{DS}}_{m_1} - {\mathrm{DS}}_{m_2}$$

which is just the difference between the discrimination slopes of the two models.

#### Net reclassification improvement (NRI)

The NRI metric, similarly to the IDI metric, measures the improvement in the discrimination of one model over another model. Following Pencina et al.^16^, suppose that we divide the unit range [0,1] into a collection of intervals or categories (for example, the intervals [0,0.1], (0.1, 0.2], (0.2, 0.3], (0.3,1.0]). Given two models $m_1$ and $m_2$, we let $v_i$ for each patient *i* be +1 if model $m_2$ places patient *i* in a higher risk interval than model $m_1$, −1 if model $m_2$ places patient *i* in a lower risk interval than model $m_1$, and otherwise 0 if the two models place the patient in the same risk interval. The NRI is then defined as.

5 $${\mathrm{NRI}}_{m_1,m_2} = \frac{{\mathop {\sum }\nolimits_{i \in S_ + } v_i}}{{N_ + }} - \frac{{\mathop {\sum }\nolimits_{i \in S_ - } v_i}}{{N_ - }}$$

Some papers refer to this NRI as the categorical NRI, as it is defined with respect to a finite collection of categories. A special case of this NRI metric is obtained when one considers an infinite continuous collection of risk categories, so that each patient is given their own category. In this case, $v_i$ for patient *i* will simply be +1 if model $m_2$ assigns a higher risk than model $m_1$, 0 if the two models assign the same risk, and −1 if model $m_2$ assigns a lower risk than $m_1$. This type of NRI is sometimes referred to as continuous NRI.

#### Net benefit (NB) and decision curves

The NB metric aims to account for the benefit from making a correct positive prediction, while accounting for the harm caused by an incorrect positive prediction^17^. To define the NB metric, one first defines a probability cutoff $\bar p$ for classifying a patient as a readmission. One then calculates the net benefit as

6 $${\mathrm{NB}} = \frac{{{\mathrm{TP}}}}{N} - \frac{{{\mathrm{FP}}}}{N} \times \frac{{\bar p}}{{\left( {1 - \bar p} \right)}}$$

where ${\mathrm{TP}}$ is the number of true positives when one uses the cutoff of $\bar p$, ${\mathrm{FP}}$ is the number of false positives from the same cutoff, and $\bar p/(1 - \bar p)$ is the exchange rate between true positives and false positives (i.e., the value of a false-positive relative to a true positive). A key feature of the net benefit metric is that the cutoff $\bar p$ that is used to classify patients as true and false positives is the same cutoff used in defining the exchange rate.

Using the concept of net benefit, one can plot a decision curve, which is a plot of net benefit against the chosen cutoff $\bar p$^17^. One can use the decision curve of a single model to find cutoff values for which the model leads to more good than harm. One can also compare the decision curves of multiple models to identify which models perform better than others.

#### Comparison with our proposed approach

The metrics and methods of evaluation that we have discussed here are quite different from the simulation-based metrics that we have proposed earlier in this section. In particular, with regard to AUROC, calibration slope and intercept, IDI and NRI, we note that these metrics are static metrics. In particular, there is no notion of time in these metrics: they are computed without accounting for when patients complete surgery and when they are discharged. They also do not account for the capacity constraint of the provider and the provider’s schedule. Our metrics, in contrast, are computed via a simulation that incorporates the provider’s schedule and the times when the patients complete surgery and are discharged. Lastly, it is also clear that these existing predictive metrics (AUROC, calibration slope and intercept, NRI and IDI) do not incorporate costs and the financial benefit of correctly intervening on true positive patients, which are incorporated in our financial metrics (ERC, ERCS, PC, and ENCS defined earlier).

With regard to the net benefit metric (and the associated concept of a decision curve), we note that this metric is closer in spirit to the simulation-based metrics that we propose here, as the net benefit metric does attempt to incorporate a “cost”, which is the cost of intervening on a false-positive patient. However, the NB metric still differs from our metrics because, like the other metrics we have highlighted above, it still does not incorporate how patients become available over time and the limit of how many patients can be seen by the provider. In addition, the meaning of a cost is different between our simulation model and the net benefit framework; in our simulation model, the cost arises from the actual financial cost of paying the provider to work and see patients, whereas, in the net benefit metric, the cost arises from incorrectly treating a false-positive patient.

We also note that the use of a cutoff probability for deciding who receives an intervention is not efficient when patients are seen by the provider dynamically. To understand why, suppose that the provider sees patients according to a particular schedule, with a fixed capacity in terms of how many patients can be seen on each day in that schedule, and only sees patients whose risk exceeds a given cutoff. On some days, we may have few or no patients who exceed the cutoff, and some patients who are below the cutoff; if the provider has capacity remaining after seeing those patients who exceed the cutoff, then it makes sense for the provider to also see some of those patients who are below the cutoff. On other days, we may have more patients who exceed the cutoff than the provider’s capacity; in this case, within the group of patients who exceed the cutoff, it makes sense to prioritize patients according to their predicted risk.

In addition, another difference between the NB metric and our proposed metrics is that the cost in the net benefit framework is defined implicitly using the chosen cutoff probability, and is a variable cost that depends linearly on the number of false-positive patients that arise from the chosen cutoff probability. In contrast, in our cost-based metrics (ERC, ERCS, PC, and ENCS), the cost is defined explicitly in dollar terms. In addition, the cost does not depend on the number of patients that the provider intervenes on: the cost only depends on the length of the simulation period, as this defines how much compensation is required for the provider.

### Generalization to other applications

The methodology we have described is explicitly formulated in terms of surgical readmissions. However, as noted earlier in the Introduction, our methodology is applicable whenever (1) there is a constrained resource that can prevent (perfectly or imperfectly) some type of event; (2) one has access to a model for predicting a given patient’s risk of that event; and (3) one is interested in quantifying the benefit of allocating the resource using the model. Our simulation model is most valuable in the early stages of model development, when one is comparing different models before committing to a single model, and reliance on classical metrics, such as AUROC, can be misleading. The main factor present in our model that is not fully addressed by the model and would need to be addressed separately is the design of the prevention resource and the prevention pathway. As discussed in the “Limitations” subsection of the “Discussion” section, our simulation model does not prescribe how the resource is able to prevent the event. As this simulation model is intended to be a stepping stone to actual real-time implementation of a machine learning-guided prevention resource, it is necessary to have already designed such a prevention resource and a pathway for effecting the prevention. Our simulation model takes the existence of such a prevention resource as a given, and does not directly suggest what the prevention resource and pathway should be. To provide more clarity on the generality of the simulation model, we describe two other potential applications of this type of approach below.

#### Sepsis prevention

Suppose that a hospital forms an intervention team that can be applied to an intensive care unit (ICU) patient to reduce that patient’s risk of sepsis, and that the team can only be applied to at most one patient each day. Suppose that the hospital develops a machine learning model for predicting the risk of sepsis for ICU patients; a number of examples of such models exist in the literature^35–37^. If one allocates the intervention team daily to the patient receiving the highest risk prediction from the model, how many cases of sepsis would be hypothetically prevented and what would be the corresponding net benefit in terms of cost? Our simulation model can be used to answer these questions.

#### Acute kidney injury prevention

Suppose that a hospital develops a machine learning model for predicting a patient’s risk of acute kidney injury^38,39^ and uses the machine learning model to automatically direct a specialist nephrologist or rapid response team to the five highest risk patients each day^40^. How many cases of AKI could be hypothetically prevented through such an ML-guided consultation scheme and how much of a reduction in cost to the hospital can be achieved through such prevention, net of the cost of the specialist/rapid response team? Our simulation model can again be used to answer these questions.

### Reporting summary

Further information on research design is available in the Nature Research Reporting Summary linked to this article.

## Supplementary information

Supplementary Information

Reporting Summary

## Data Availability

The data sets generated during and/or analyzed during the current study are not publicly available due to institutional restrictions on data sharing and privacy concerns. However, the data are available from the authors on reasonable request.

## Code availability

The code used to perform our simulations was written in Julia version 1.5.0^31^ and is available at http://github.com/vvmisic/finsim-code/.
