# Supplementary material for: A simulation-based evaluation of machine learning models for clinical decision support: application and analysis using hospital readmission
Source: NPJ Digit Med. 2021 Jun 14;4:98. doi: 10.1038/s41746-021-00468-7 (PMC8203794; doi:10.1038/s41746-021-00468-7)
Supplement: Supplementary file 1 — Supplementary Information [file 41746_2021_468_MOESM1_ESM.pdf]

Supplementary Table 1: Summary of surgical patient characteristics at UCLA RRMC from 2017 to 2018.

Admissions	19331
Admissions with unplanned emergency department readmission	969 (5.0%)
Length of stay (days)	6 +/- 13
Age (years)	54 +/- 18
ASA Score:	
1	1139 (5.9%)
2	6628 (34.3%)
3	9479 (49.0%)
4	1776 (9.2%)
5	113 (0.6%)
NA	196 (1.0%)
Admissions with primary DRG code	12469 (64.5%)
Number of surgical CPT codes	2 +/- 2
Number of DRG codes	10 +/- 8
Number of readmissions in preceding year	1 +/- 1
Surgical information*:	
Total blood loss (mL)	285 +/- 1216
Total fluids transfused (mL)	1110 +/- 807
Total colloid transfused (mL)	636 +/- 531
Total blood transfused (mL)	105 +/- 847
Duration of anesthesia (minutes)	250 +/- 163
Hemodialysis	350 (1.8%)
Tracheostomy (number of admissions)	346 (1.8%)
Mechanical ventilation (number of admissions)	2228 (11.5%)
Duration of mechanical ventilation* (minutes)	6081 +/- 13256
Disposition:	
Home or self care	14074 (72.8%)
Home health service	3532 (18.3%)
Skilled nursing facility	668 (3.5%)

Other	1057 (5.5%)
Surgical Service Lines	
Cardiac Surgery	1455 (7.5%)
General Surgery	3407 (17.6%)
Liver Transplant	563 (2.9%)
Neurosurgery	2055 (10.6%)
Obstetrics and Gynecology	1199 (6.2%)
Ophthalmology	216 (1.1%)
Oral and Maxillofacial Surgery	308 (1.6%)
Orthopaedics	1182 (6.1%)
Other	120 (0.6%)
Otolaryngology	2829 (14.6%)
Pediatric Surgery	68 (0.4%)
Plastic Surgery	918 (4.7%)
Radiation Oncology	103 (0.5%)
Radiology	2 (0.0%)
Surgical Oncology	535 (2.8%)
Thoracic Surgery	792 (4.1%)
Urology	2557 (13.2%)
Vascular Surgery	1022 (5.3%)

Table Legend: Values are reported as either “number of admissions (% of total data set)” or as “mean +/- standard deviation”. For variables or groups of variables marked with *, the mean and standard deviation are taken over observations with non-missing values for those variables.

Supplementary Table 2: Patient performance metrics for the four different models under different effectiveness constants.

Schedule	Capacity	Method	PS	RA	ERP (5%)	ERP (10%)	ERP (20%)
M	8	L1LR (with labs)	866	258	12.9	25.8	51.6
M	8	L1LR (no labs)	861	155	7.8	15.5	31.0
M	8	LACE	845	91	4.6	9.1	18.2
M	8	HOSPITAL	845	86	4.3	8.6	17.2
M W	8	L1LR (with labs)	1705	423	21.2	42.3	84.6
M W	8	L1LR (no labs)	1699	263	13.2	26.3	52.6
M W	8	LACE	1688	178	8.9	17.8	35.6
M W	8	HOSPITAL	1688	173	8.7	17.3	34.6
M T W R F	8	L1LR (with labs)	4199	672	33.6	67.2	134.4
M T W R F	8	L1LR (no labs)	4196	502	25.1	50.2	100.4
M T W R F	8	LACE	4215	456	22.8	45.6	91.2
M T W R F	8	HOSPITAL	4215	437	21.9	43.7	87.4

Table Legend: Under "Method", "L1 LR" denotes L1 regularized logistic regression. "Schedule" indicates the days of the week on which the provider works (M = Monday, T = Tuesday, W = Wednesday, R = Thursday, F = Friday). For the last four columns, PS denotes the number of patients seen (how many patients were seen by the provider over the simulation horizon); RA denotes the number of readmissions anticipated (how many patients were seen by the provider and had an ER readmission); ERP denotes the expected readmissions prevented (RA multiplied by the effectiveness coefficient, assumed to be 10%); and RLOS denotes the readmission length of stay (sum of the LOS at readmission of those patients who were selected and who had an ER readmission).

Supplementary Table 3: Cost performance metrics for the four different models under different effectiveness constants.

Schedule	Method	ERCS @ 5%	ENCS @ 5%	ERCS @ 10%	ENCS @ 10%	ERCS @ 20%	ENCS @ 20%
M	L1LR (with labs)	187,295	114,695	374,590	301,990	749,180	676,580
M	L1LR (no labs)	111,600	39,000	223,200	150,600	446,400	373,800
M	LACE	66,045	(6,555)	132,090	59,490	264,180	191,580
M	HOSPITAL	62,620	(9,980)	125,240	52,640	250,480	177,880
M W	L1LR (with labs)	307,135	161,935	614,270	469,070	1,228,540	1,083,340
M W	L1LR (no labs)	189,535	44,335	379,070	233,870	758,140	612,940
M W	LACE	128,860	(16,340)	257,720	112,520	515,440	370,240
M W	HOSPITAL	125,665	(19,535)	251,330	106,130	502,660	357,460
M T W R F	L1LR (with labs)	488,800	35,050	977,600	523,850	1,955,200	1,501,450
M T W R F	L1LR (no labs)	363,275	(90,475)	726,550	272,800	1,453,100	999,350
M T W R F	LACE	330,920	(122,830)	661,840	208,090	1,323,680	869,930
M T W R F	HOSPITAL	317,540	(136,210)	635,080	181,330	1,270,160	816,410

Table Legend: Under "Method", "L1 LR" denotes L1 regularized logistic regression. "Schedule" indicates the days of the week on which the provider works (M = Monday, T = Tuesday, W = Wednesday, R = Thursday, F = Friday). The columns report the expected readmission cost savings (ERCS) and expected net cost savings (ENCS) at different effectiveness constants (5%, 10%, 20%). Note that 10% corresponds to the base case shown in Tables 2 and 3. The capacity, provider cost (PC) and expected readmission cost (ERC) are the same as in Table 3, and are omitted to facilitate presentation. Parenthesized quantities in red font correspond to negative values (i.e., expected net cost savings that are negative, where the provider cost exceeds the expected readmission cost savings).

Supplementary Table 4: Cost performance metrics for the four different models under different provider costs.

Schedule	Method	PC (0.5x)	ENCS (0.5x)	PC (1x)	ENCS (1x)	PC (2x)	ENCS (2x)
M	L1LR (with labs)	36,300	338,290	72,600	301,990	145,200	229,390
M	L1LR (no labs)	36,300	186,900	72,600	150,600	145,200	78,000
M	LACE	36,300	95,790	72,600	59,490	145,200	(13,110)
M	HOSPITAL	36,300	88,940	72,600	52,640	145,200	(19,960)
M W	L1LR (with labs)	72,600	541,670	145,200	469,070	290,400	323,870
M W	L1LR (no labs)	72,600	306,470	145,200	233,870	290,400	88,670
M W	LACE	72,600	185,120	145,200	112,520	290,400	(32,680)
M W	HOSPITAL	72,600	178,730	145,200	106,130	290,400	(39,070)
M T W R F	L1LR (with labs)	226,875	750,725	453,750	523,850	907,500	70,100
M T W R F	L1LR (no labs)	226,875	499,675	453,750	272,800	907,500	(180,950)
M T W R F	LACE	226,875	434,965	453,750	208,090	907,500	(245,660)
M T W R F	HOSPITAL	226,875	408,205	453,750	181,330	907,500	(272,420)

Table Legend: Under "Method", "L1 LR" denotes L1 regularized logistic regression. "Schedule" indicates the days of the week on which the provider works (M = Monday, T = Tuesday, W = Wednesday, R = Thursday, F = Friday). The columns report the provider cost (PC) and expected net cost savings (ENCS) under three different scenarios for provider cost (the provider cost is 0.5, 1 or 2 times the provider cost in the base case). Note that "1x" corresponds to the base case in Table 3. The capacity, expected readmission cost and expected readmission cost savings are the same as in Table 3, and are omitted to facilitate presentation. Parenthesized quantities in red font correspond to negative values (i.e., expected net cost savings that are negative, where the provider cost exceeds the expected readmission cost savings).

Supplementary Table 5: Cost performance metrics for the four different models under alternate provider cost model.

Schedule	Method	PC ($100/pat)	ENCS ($100/pat)	PC ($200/pat)	ENCS ($200/pat)	PC ($300/pat)	ENCS ($300/pat)
M	L1LR (with labs)	86,600	287,990	173,200	201,390	259,800	114,790
M	L1LR (no labs)	86,100	137,100	172,200	51,000	258,300	(35,100)
M	LACE	84,500	47,590	169,000	(36,910)	253,500	(121,410)
M	HOSPITAL	84,500	40,740	169,000	(43,760)	253,500	(128,260)
M W	L1LR (with labs)	170,500	443,770	341,000	273,270	511,500	102,770
M W	L1LR (no labs)	169,900	209,170	339,800	39,270	509,700	(130,630)
M W	LACE	168,800	88,920	337,600	(79,880)	506,400	(248,680)
M W	HOSPITAL	168,800	82,530	337,600	(86,270)	506,400	(255,070)
M T W R F	L1LR (with labs)	419,900	557,700	839,800	137,800	1,259,700	(282,100)
M T W R F	L1LR (no labs)	419,600	306,950	839,200	(112,650)	1,258,800	(532,250)
M T W R F	LACE	421,500	240,340	843,000	(181,160)	1,264,500	(602,660)
M T W R F	HOSPITAL	421,500	213,580	843,000	(207,920)	1,264,500	(629,420)

Table Legend: Under "Method", "L1 LR" denotes L1 regularized logistic regression. "Schedule" indicates the days of the week on which the provider works (M = Monday, T = Tuesday, W = Wednesday, R = Thursday, F = Friday). The columns report the provider cost (PC) and expected net cost savings (ENCS) under an alternate provider cost model, where there is a cost to each patient seen by the provider (the six columns correspond to three scenarios for this variable cost: $100 per patient seen, $200 per patient seen and $300 per patient seen). The provider cost is calculated by multiplying the PS metric by each of the three per-patient costs. The capacity, expected readmission cost and expected readmission cost savings are the same as in Table 3, and are omitted to facilitate presentation. Parenthesized quantities in red font correspond to negative values (i.e., expected net cost savings that are negative, where the provider cost exceeds the expected readmission cost savings).
